# Keggin-Type Heteropoly Salts as Bifunctional Catalysts in Aerobic Baeyer-Villiger Oxidation

**DOI:** 10.3390/ma11071208

**Published:** 2018-07-13

**Authors:** Katarzyna Pamin, Jan Połtowicz, Mateusz Prończuk, Joanna Kryściak-Czerwenka, Robert Karcz, Ewa M. Serwicka

**Affiliations:** 1 Jerzy Haber Institute of Catalysis and Surface Chemistry, Polish Academy of Sciences, Niezapominajek 8, 30-239 Krakow, Poland; ncpoltow@cyf-kr.edu.pl (J.P.); nckrysci@cyf-kr.edu.pl (J.K.-C.); nckarcz@cyf-kr.edu.pl (R.K.); ncserwic@cyf-kr.edu.pl (E.M.S.); 2 Faculty of Chemical Engineering and Technology, Cracow University of Technology, Warszawska 24, 31-155 Krakow, Poland; mpronczuk@chemia.pk.edu.pl

**Keywords:** polyoxometalates, Baeyer-Villiger oxidation, ε-caprolactone, molecular oxygen

## Abstract

The cobalt, manganese, and iron salts of tungstophosphoric or molybdophosphoric acid with growing content of metals were applied for the first time as catalysts in the Baeyer-Villiger (BV) oxidation of cyclohexanone to ε-caprolactone with molecular oxygen. The catalysts were characterized with Fourier transform infrared spectroscopy (FTIR), X-ray fluorescence (XRF), and ethanol decomposition reaction. Introduction of transition metals into the heteropoly structure increases the activity of resulting heteropoly salts in comparison with parent heteropolyacids. It was shown that the most active catalysts are salts of the heteropoly salts with one metal atom introduced and one proton left (HMPX) type, (where M = Co, Fe, Mn, and X = W, Mo) with the metal to proton ratio equal one. Among all of the studied catalysts, the highest catalytic activity was observed for HCoPW. The effect indicates that both the acidic and redox properties are required to achieve the best performance. The Baeyer-Villiger (BV) oxidation mechanism proposed identifies the participation of heteropoly compounds in three steps of the investigated reaction: oxidation of aldehyde to peracid (redox function), activation of carbonyl group (Lewis acidity), and decomposition of the Criegee adduct to ε-caprolactone (Brønsted acidity).

## 1. Introduction

Heteropoly compounds have attracted great interest as catalysts because their acidic and redox functions can be tuned at the molecular level by appropriate choice of the constituting elements. The most widely studied heteropoly compounds are based on Keggin anions. A typical structure of Keggin anion is represented by formula (XM_12_O_40_)^n−^, where X denotes the central heteroatom contained in a tetrahedron, and M is the addenda atom, usually Mo(VI) or W(VI), placed in a MO_6_ octahedron [1]. Modification of heteropolyacids with selected transition metal ions makes them effective catalysts of oxidation reactions [2]. The addition of such dopants offers means of controlling the catalysts multifunctionality, by the modification of their acid-base and redox properties. In addition, polyoxometalates are stable over a wide range of conditions and are resistant to oxidative degradation, which ensures an extended lifetime in oxidation reactions.

The Baeyer-Villiger (BV) oxidation is a method for converting cyclic ketones into lactones or ketones into esters. The products of the BV oxidation find a wide area of possible applications as pharmaceuticals (i.e., antibiotics or steroids), monomers for biodegradable plastics, or intermediates for fine chemicals [3].

The most common oxidants that are applied in this reaction are peroxycarboxylic acids, but due to their hazardous nature there is an ever increasing interest in application of more ecologically friendly reagents, such as hydrogen peroxide or dioxygen in the presence of sacrificial aldehyde [4].

The advantage of hydrogen peroxide is its low cost and the formation of water as the only by-product. Among many catalysts tested in BV processes with H_2_O_2_ as oxidant, were heteropolyacids and their salts [5,6,7]. Thus, in the presence of H_3_PW_12_O_40_, cyclic ketones can be oxidized to appropriate lactones with moderate selectivity [5]. The Cs_2.5_H_0.5_PW_12_O_40_ salt, due to its specific surface structure, demonstrated higher selectivity to ε-caprolactone than the parent heteropolyacid H_3_PW_12_O_40_ [6]. H_3_SiW_12_O_40_ supported on multi-walled carbon nanotubes showed high catalytic activity in BV oxidation of a series of cyclic ketones. Heterogenization of tungstosilicic acid enabled an easy recovery and reuse of the catalyst in cyclopentanone oxidation [7]. However, there are also some disadvantages that are associated with the use of hydrogen peroxide, such as instability at higher concentrations, which may cause a build-up of oxygen pressure, which is potentially dangerous in combination with flammable organics [8].

**Figure 1:**
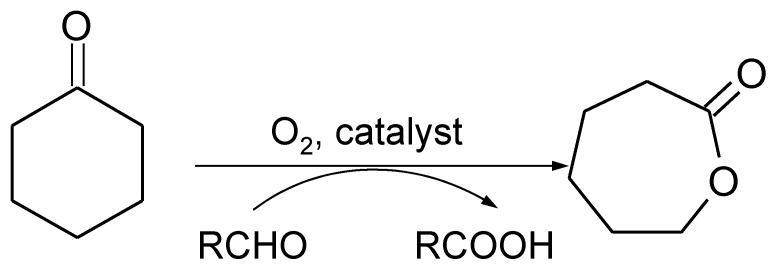
Scheme of catalytic Baeyer-Villiger (BV) cyclohexanone oxidation with molecular oxygen and sacrificial aldehyde—Mukaiyama system.

In 1991, Mukaiyama developed a catalytic system [9] that enables BV transformation to be carried out over Ni complexes in the presence of molecular oxygen and sacrificial aldehyde (Figure 1). These conditions are considered an attractive alternative, since molecular oxygen is an inexpensive, environmentally friendly, and easily available oxidant [10]. This type of BV oxidation proceeds effectively in the presence of metal catalysts via free radical chain mechanism.

There exists a rich literature on the application of Mukaiyama conditions to BV oxidation. Raja et al. [10] demonstrated that redox molecular sieve catalysts MAlPO-36 (M = Mn or Co) convert cyclopentanone, cyclohexanone, 2-methylcyclohexanone, and adamantan-2-one to their corresponding lactones with high efficiency in the presence of O_2_ and benzaldehyde as sacrificial oxidant. A number of works observed high activity of Fe-containing catalysts. Thus, Fe_2_O_3_ proved to be excellent catalyst of oxidation of cyclic ketones to lactones in the presence of benzaldehyde [11]. Interestingly, other Fe-based catalysts, such as iron powder, hydrated FeCl_2,_ and Fe(OAc)_3_ were inactive in the studied reaction. Lakk-Bogath et al. [12] reported that high-valent oxoiron species were active in the oxidation of cyclohexanone derivatives with benzaldehyde as sacrificial co-reductant. Another catalytic system, Fe–Sn–O mixed oxides, performed very well in the oxidation of cyclohexanone in the presence of benzaldehyde, but its activity in the case of aliphatic ketones was poorer [13]. Iron containing cubic mesoporous MCM-48 materials, which were used as catalysts in Mukaiyama system with benzaldehyde, proved to be very active and showed excellent recyclability [14]. Recently, Cu, Fe, and Ni promoted cobalt containing mesoporous silicas were reported as catalytically active system for BV oxidation of cyclohexanone, in the presence of benzaldehyde [15]. Surprisingly, hardly any works addressed BV oxidation in the Mukaiyama system over polyoxometalates as catalysts. In the paper that was published in 1993, Hamamoto et al. [16] used a complex mixture consisting of Keggin-type polyoxometalates, with the secondary structure involving vanadium cations, for BV oxidation of cyclic and aliphatic ketones under Mukaiyama conditions, and observed that the reaction was much more efficient in the case of cyclic ketones. The authors suggested that, with aliphatic ketones as substrates, the perbenzoic acid, generated from oxygen and benzaldehyde, oxidizes benzaldehyde itself rather than the ketone, to form benzoic acid in preference to the Baeyer-Villiger product. Recently, we have reported on the catalytic activity of a new hybrid porphyrin-heteropolyacid material and some heteropolysalts of transition metals in the aerobic BV oxidation of cyclohexanone in the presence of sacrificial propionaldehyde [17]. Both types of heteropoly compounds showed enhanced activity in comparison with the parent heteropolyacids. Of interest are more recent attempts to use enzymes as regio- and stereo-selective catalysts for BV transformation [18,19,20]. However, the implementation of this strategy is still a challenge, mainly because of limited enzyme stability, and the costs of catalysts production.

In the present work we undertook a more detailed study of the effect of counter-cations in heteropoly salts on the catalytic performance in BV oxidation under the Mukaiyama regime. It was expected that the cationic species would act as additional functionalities, both redox, through their ability to assume various oxidation states and acid/base, via the ability to act as Lewis acid centers themselves, or Brønsted acid centers via the properties of coordinated water of hydration. We describe the application of cobalt, manganese, and iron as compensating cations in the Keggin type of heteropolyanions to the liquid-phase BV oxidation of cyclohexanone to ε-caprolactone in the oxygen-aldehyde system. Two series of catalysts were investigated: one based on tungstophosphoric (H_3_PW), the other on molybdophosphoric (H_3_PMo) acid. In each series, salts with three degrees of proton for transition metal cation substitution were prepared. The general formula of the samples is H_3−2x_M_x_PX_12_O_40_, where x = ½, 1, 1 ½, M = Co, Mn, Fe, and X = Mo or W. The catalytic performance of heteropoly salts is compared with that of parent heteropolyacids.

## 2. Materials and Methods

### 2.1. Materials

Cobalt and manganese dodecatungstophosphate or dodecamolybdophosphate were synthesized as precipitates by mixing an aqueous solution of an appropriate amount of cobalt(II) or manganese(II) carbonate (Aldrich, Steinheim, Germany) with an aqueous solution of selected heteropolyacid. Detailed preparative procedure has been described in [21]. Iron dodecatungstophosphate or dodecamolybdophosphate salts were synthesized by adding the stoichiometric amount of FeCl_2_ to an aqueous solution of suitable heteropolyacid. The solution was filtered and the resulting precipitate was evaporated to dryness in the oven at 90 °C. The following salts with varying number of metal cation in the heteropoly structure were synthesized: H_2_Co_0.5_PW_12_O_40_, HCoPW_12_O_40_, Co_1.5_PW_12_O_40,_ H_2_Co_0.5_PMo_12_O_40_, HCoPMo_12_O_40_, Co_1.5_PMo_12_O_40_, H_2_Mn_0.5_PW_12_O_40_, HMnPW_12_O_40_, Mn_1.5_PW_12_O_40_, H_2_Mn_0.5_PMo_12_O_40_, HMnPMo_12_O_40_, Mn_1.5_PMo_12_O_40_, H_2_Fe_0.5_PW_12_O_40_, HFePW_12_O_40_, Fe_1.5_PW_12_O_40,_ H_2_Fe_0.5_PMo_12_O_40_, HFePMo_12_O_40_, Fe_1.5_PMo_12_O_40_ and for simplicity designated as H_2_Co_0.5_PW, HCoPW, Co_1.5_PW, H_2_Co_0.5_PMo, HCoPMo, Co_1.5_PMo, H_2_Mn_0.5_PW, HMnPW, Mn_1.5_PW, H_2_Mn_0.5_PMo, HMnPMo, Mn_1.5_PMo, H_2_Fe_0.5_PW, HFePW, Fe_1.5_PW, H_2_Fe_0.5_PMo, and HFePMo, Fe_1.5_PMo, respectively. The acid formulae are abbreviated H_3_PW and H_3_PMo.

### 2.2. Methods

Chemical composition of the Keggin anions (W, Mo, P, Mn, Fe, Co) was determined by means of the X-ray fluorescence (XRF), with an Orbis Micro EDXRF spectrophotometer, 00using 30 kV radiation.

FTIR spectra were recorded on a Nicolet 6700 spectrometer under atmospheric conditions. Spectra were recorded in range of 4000–400 cm^−1^ with resolution of 2 cm^−1^ and collecting 64 scans. FTIR study of pyridine adsorption (POCh Gliwice, Gliwice, Poland, analytical grade, dried over 3A molecular sieve) was carried out using self-supporting pellets of the catalysts, placed in a quartz cell equipped with CaF_2_ windows, and designed to perform measurements at different temperatures. Prior to pyridine adsorption, the sample was outgassed at 200 °C under vacuum for 1 h. Then, the cell was cooled to room temperature and the spectrum of activated sample was obtained. Later, the sample was allowed to interact with pyridine at room temperature. Thereafter, the cell was outgassed for 30 min under vacuum at 150 °C and 200 °C. After cooling down, FTIR spectra of the samples were measured. For each measurement 64 scans were taken with a resolution of 2 cm^−1^ using a Nicolet 710 FTIR spectrometer. The amounts of Brønsted and Lewis acid sites were estimated on the basis of the band intensities around 1540 and 1450 cm^−1^, respectively, as described by Emeis [22].

The decomposition of ethanol was carried out in a conventional flow type reactor under atmospheric pressure. Typically, a 0.3 mL sample was placed in a quartz reactor. Ethyl alcohol was introduced into the helium stream (5.7% mole in He) by the thermostated evaporator-saturator set. The total flow rate of alcohol in the stream of the feed gas was 1.8 L/h. Before the test the catalyst was heated to 300 °C at the rate 100 K/h and kept in a helium flow for 1 h. Catalytic tests were run at 250 °C. The products were analyzed by means of the Perkin-Elmer 900 gas chromatograph equipped with FID detector and Porapak S column.

The Baeyer-Villiger oxidation of cyclohexanone with molecular oxygen was performed in a thermostated home-made glass reactor at 40 °C for 5 h at atmospheric pressure. In a typical experiment, 0.01 mM of a catalyst, 4.6 mmol of cyclohexanone, and 14 mmol of aldehyde were dissolved in 10 mL of acetonitrile. Under these conditions, the catalysts are dissolved in acetonitrile and form homogeneous liquid phase with the reactants. In the course of the reaction, the concentration of oxygen was kept constant with aid of the valves system controlling the level of oxygen in the reaction mixture. The conversion of substrate (percent of consumed cyclohexanone) and the percentage yield of ε-caprolactone (calculated as the amount of desired product obtained divided by the theoretical yield predicted by the reaction stoichiometry) were determined using Agilent Technologies 6890 N gas chromatograph equipped with a FID detector and Innowax (30 m) column, in the presence of chlorobenzene as internal standard. Gas chromatograph (GC) analysis was performed in the programmed oven method with the temperature rise from 40 to 170 °C and heating speed of 20 °C/min. In the conditions of GC analysis, the retention times for cyclohexanone, chlorobenzene, and ε-caprolactone were detected as 7.57 min, 12.18 min, and 14.40 min, respectively. GC parameters were quantified in calibration by the standard samples prior to the analysis. The percentage yield was calculated as the amount of desired product obtained, divided by the theoretical yield, and predicted by the stoichiometric calculation.

## 3. Results and Discussion

The materials were synthesized according to the established procedures, developed over the years since the work of Tsigdinos [23]. However, in view of the known hydrolytic lability of Keggin anions, XRF and FTIR have been used as chief tools to ascertain the chemical identity of the catalyst, some of the data having been discussed in our previous works [21,24].

Results of XRF measurements of the investigated materials, as presented in Table 1, show that the contents of key elements constituting the synthesized heteropoly compounds are close to the values expected from the stoichiometric formulae of the samples.

FTIR spectroscopy is an established tool for the structural characterization of heteropoly acids and their salts, and it provides vital information on the chemical identity of the synthesized materials. FTIR spectra of all catalysts are shown in Supplementary Materials (Figures S1–S3). Keggin unit displays a characteristic fingerprint of four bands in the 600–1100 cm^−1^ range of the infrared spectrum [25]. The bands are attributed, in the order of decreasing wavenumbers, to the asymmetric stretching modes of P–O_a_ (1050–1080 cm^−1^), M–O_d_ (960–1000 cm^−1^), M–O_b_ (870–900 cm^−1^), and M–O_c_ (780–810 cm^−1^) bonds, where O_a_—inner oxygen atom bonded to the central heteroatom, O_b_—bridging oxygen atom bonding two metal centers in adjacent corner-sharing octahedra, O_c_—bridging oxygen atom bonded to two metal centers in adjacent edge-sharing octahedra and O_d_—terminal oxygen atom attached to only one metal center. The band positions and/or intensities are sensitive to the modification of the heteropoly compound composition and/or structure, in particular, to the symmetry changes that are associated with the structural alteration accompanying the formation of lacunary species or their derivatives [2,21,24,25,26,27]. Results of FTIR analysis of synthesized materials are consistent with the desired compositions of the solids. In particular, there is no indication of splitting of P–O stretching band, as expected in the case of lacunary anion formation, and resulting from the lowering of the overall symmetry of the Keggin anion from T_d_ to C_s_ upon the hydrolytic removal of one addenda center, nor a shift of this band, which would occur if vacancies in lacunary anions would be filled with transition metal cation other than the parent W or Mo. Thus, the FTIR study shows that the synthesized compounds do not contain lacunary anions, or derivatives of lacunary anions, in amounts, which could impact the obtained data. Within the accuracy of XRF and sensitivity of FTIR the materials contain intact Keggin anions and counter cations in intended quantities, therefore the observed catalytic dependencies can be related the intended compositions.

Assessment of acid-base and redox functions can be carried out by means of the catalytic test of ethanol decomposition. In the presence of acid centers, alcohol is transformed into diethyl ether and ethylene, while over redox centers it is converted via oxidative dehydrogenation into acetaldehyde. Results of such a test are presented in Table 2. Analysis of the data leads to the general conclusion that the W-based series displays strong acidic properties, which fall upon the substitution of protons with metal ions. No formation of acetaldehyde is observed, indicating that inserted transition metal ions in combination with 12-tungstophgosphate anion do not produce strong redox sites. Series that are derived from molybdophosphoric acid shows weaker acidic properties, diminishing further with an increasing content of transition metal cations. Simultaneously, formation of acetaldehyde points to the presence of much more efficient redox centers than in the case of W-based catalysts. Selectivity to acetaldehyde increases upon growing replacement of protons with metal cations, the effect that is attributed to the suppression of the acid function.

**Figure 2:**
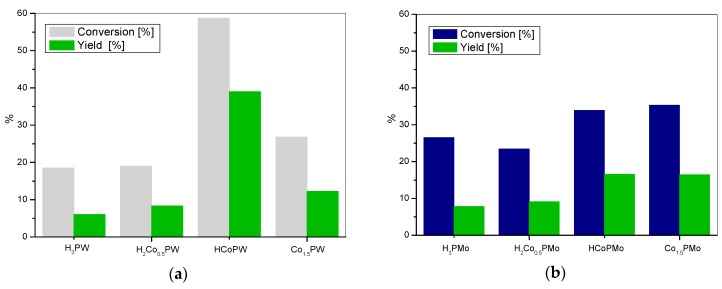
The BV oxidation of cyclohexanone with (**a**) cobalt tungstophosphate catalysts and (**b**) cobalt molybdophosphate catalysts.

**Figure 3:**
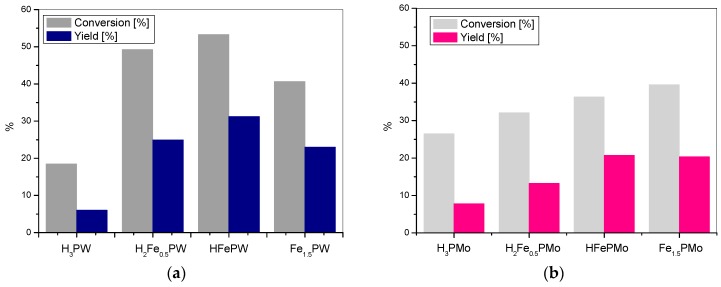
The BV oxidation of cyclohexanone with (**a**) iron tungstophosphate catalysts and (**b**) iron molybdophosphate catalysts.

**Figure 4:**
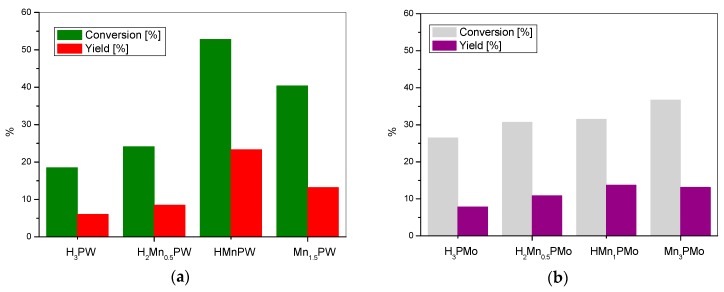
The BV oxidation of cyclohexanone with (**a**) manganese tungstophosphate catalysts and (**b**) manganese molybdophosphate catalysts.

The synthesized catalysts were applied in the Baeyer-Villiger oxidation of cyclohexanone with molecular oxygen to ε-caprolactone. Figure 2, Figure 3 and Figure 4 present the results of catalytic activity in the studied reaction for cobalt, manganese, or iron tungstophosphates and molybdophosphates, together with the data for the appropriate parent heteropolyacid. The catalytic activity of cobalt tungstophosphates and molybdophosphates is shown in Figure 2.

It may be seen that H_3_PW displays the lowest catalytic activity within the studied W-based series (Figure 2a). For the catalyst with the lowest content of cobalt, H_2_Co_0.5_PW, a slight improvement of ε-caprolactone yield is found. In the case of the catalyst with two protons substituted for one cobalt atom (HCoPW), a very significant increase of catalytic activity is observed and the yield of ε-caprolactone approaches 40%. Finally, the activity of the neutral Co_1.5_PW salt drops in comparison to the HCoPW complex. The occurrence of an optimum composition suggests that more than one factor influences the course of the reaction. Initially, the addition of cobalt cations is clearly beneficial. Co centers are known to be able to activate dioxygen, therefore their redox properties are essential for oxidation of the sacrificial aldehyde to peracid. However, the drop of activity at maximum Co content indicates that Co-rich catalyst lacks some other characteristics that are required for the efficient catalytic transformation. From the study of acid-base properties of the materials, it may be concluded that this missing function is the catalyst acidity, which is shown to fall upon growing substitution with Co. Cobalt molybdophosphate series is less active in BV oxidation than cobalt tungstophosphates (Figure 2b). Also here, the lowest activity towards ε-caprolactone synthesis is observed for the parent heteropolyacid, and the addition of cobalt improves the catalytic performance only up to an intermediate degree of substitution, corresponding to HCoPMo catalyst. The effect is in accordance with the postulated necessity of the optimization of catalytic redox and acid functions. Noteworthy, the differences between the members of the series are less pronounced than in the case of tungstophosphates. This may be related to much weaker acidic properties of the molybdophosphate salts, which, in addition, show lesser relative differences between the catalysts.

Introduction of iron into the heteropoly structure affects the catalytic activity of synthesized catalysts, according to the similar pattern as in the case of cobalt heteropoly salts. The growing number of iron centers results in the gradual rise of the catalytic activity up to the highest value observed for HFePW (Figure 3a) and HFePMo (Figure 3b), indicating again that the active catalyst should be bifunctional. Also, in this case variation of catalytic activity with catalyst composition is more pronounced for the tungsten series, confirming that, for the studied materials, it is the modification of the acid function that has the strongest impact on the course of reaction.

Finally, the catalytic profiles observed for the manganese tungstophosphates (Figure 4a) and manganese molybdophosphates (Figure 4b) reveal catalytic behavior that is similar to that of the previously described series.

Comparison of the catalytic data in Figure 2, Figure 3 and Figure 4 shows that, in general, the catalytic activity of the metal salts of tungstophosphoric acid is higher than the activity of their molybdophosphate analogues. It seems reasonable to attribute this effect to the much weaker acidic properties of molybdophosphates.

Trying to understand the reaction mechanism one should remember that the reaction in the Mukaiyama system is based on the easy autoxidation of aldehydes to promote the in situ formation of peroxy acids [10]. The process requires the abstraction of hydrogen from aldehyde to produce acyl radical RCO•. In the presence of unsubstituted heteropolyacid, H_3_PX hydrogen is abstracted by addenda metal X at the highest oxidation state [10,21]. In the case of heteropolysalt of Co^2+^, Mn^2+^, or Fe^2+^, an additional, more efficient path of acyl radical formation becomes possible via activation of molecular oxygen to a radical form, which, in turn, easily abstracts hydrogen from aldehyde to yield acyl species [21]. The acyl radicals react with molecular oxygen forming acylperoxy radical species. The latter abstract hydrogen from other aldehyde molecules, which leads to the in situ formation of peracid.

Basing on the literature data [10,28,29] and the results of the present study, we conclude that the differences in the catalytic activity observed between unsubstituted heteropoyacids and their cobalt, iron, and manganese heteropoly salts are related to the reaction dependence on both the redox and the acid functions, and evolution thereof upon gradual substitution of protons with transition metal cations. The chief source of the redox function, as required for the activation of oxygen, are the transition metal cations that are introduced as species compensating the charge of heteropolyanion. Cobalt and iron activate oxygen by formation of superoxo complexes with oxygen [30,31]. In the case of manganese, a peroxo complex with oxygen is postulated [32]. However, the transition metal cations may also act as Lewis acids, which, as demonstrated by Corma [29], may coordinate ketone, and thereby activate the carbonyl group. In order to check on the nature of acid sites in the studied heteropoly compounds, the H_3−2x_Co_x_PW_12_O_40_ series has been subjected to the experiment with pyridine adsorption monitored by FTIR. The results unequivocally demonstrated that, while the parent H_3_PW is characterized exclusively by Brønsted acidity, the introduction of Co cations is associated with generation of Lewis acid sites. For the pyridine adsorption/desorption experiment carried out at 150 °C, the ratio of Lewis to Brønsted acid sites was 0 for H_3_PW, 0.3 for H_2_Co_0.5_PW, 1.1 for HCoPW, and 1.7 for Co_1.5_PW. Therefore, we presume that transition metal cations also influence the reaction by contributing to the activation of the carbonyl group. Decomposition of the intermediate Criegee adduct has been shown to require the presence of protons [30]; therefore, it is assumed that Brønsted acidity is of particular importance in the final stage of the reaction.

**Figure 5:**
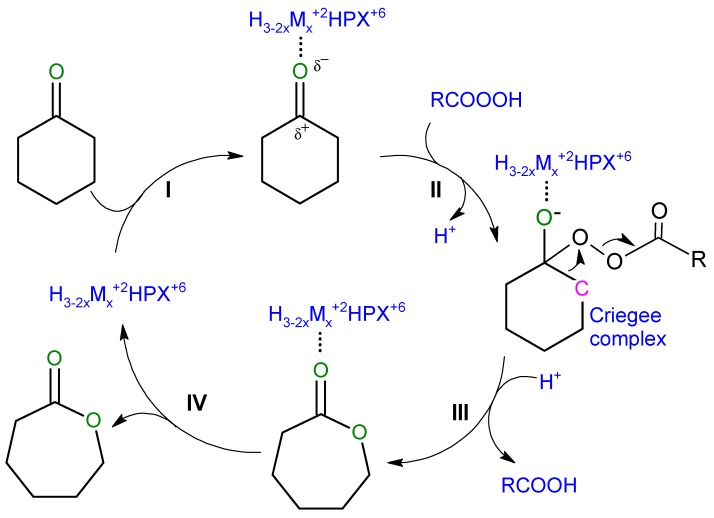
Mechanism of BV oxidation of cyclohexanone in Mukaiyama catalytic system.

In view of the above, we propose the following mechanistic scheme of the reaction (Figure 5), identifying steps in which the heteropoly catalyst containing metal cations may be involved (other than already described peracid generation). Thus, in heteropoly salt-like catalysts the cyclohexanone molecule may become coordinated to the catalyst Lewis metal center (step I), activating carbonyl group of ketone and rendering it more prone to nucleophilic attack by the incoming peracid (step II), which results in the formation of the Criegee complex [28,29].

The next step relies on the rearrangement of the Criegee complex. Dissociation of the O–O bond results in the detachment of RCOO group and the incorporation of the remaining oxygen atom into ketone structure. Detached RCOO group undergoes protonation with the formation of carboxylic acid (step III). The intermediate product-catalyst complex decomposes with the formation of ε-caprolactone and the release of the catalyst (step IV). which may further react with another substrate molecule.

The above mechanistic considerations are consistent with the experimental observations. In particular, it is shown that the beneficial role of transition metal cations is associated with (a) their participation in the oxidation of sacrificial aldehyde to peracid, and (b) possible activation of cyclohexanone on the Lewis acid sites. The scheme presented in Figure 5 underlines the role of Brønsted acidity in the final transformation of Criegee intermediate to lactone, because protons are required to complete the detachment of carboxylic acid molecule. This shows the importance of sufficient proton concentration and explains why optimum catalytic performance is observed over partially substituted heteropoly salts, whose composition offers the required bifunctionality, and why more acidic W-based catalysts perform better than their Mo-based counterparts.

## 4. Conclusions

Cobalt, manganese, and iron as compensating cations in the W- or Mo-Keggin type of heteropolyanions were applied in the liquid-phase BV oxidation of cyclohexanone to ε-caprolactone in the oxygen-aldehyde system (Mukaiyama regime). Introduction of transition metal cations into heteropoly structure creates new catalytic centers that modify the acidic and redox properties of the compound. This results in the formation of bifunctional catalysts and the alteration of catalytic activity. In general, the incorporation of metal atoms (M = Co, Fe, Mn) at the position of the compensating cation increases the catalytic activity of the resulting heteropoly salts with respect to parent heteropolyacids. However, the size of the catalytic effects depends on the type of addenda atom. Generally, catalysts of the tungstophosphate series, having stronger acidic properties, are more active and show a stronger variation of catalytic performance than those of the molybdophosphate type. As a rule, within each catalytic series, heteropoly salts with one metal atom introduced and one proton left (HMPX, where M = Co, Fe, Mn and X = W, Mo) demonstrate the highest catalytic activity in the cyclohexanone BV oxidation with molecular oxygen. The effect implies that high catalytic activity in the transformation of cyclohexanone to ε-caprolactone can be achieved for the catalyst that possesses both the acidic and redox properties.

The BV oxidation mechanism proposed identifies the participation of heteropoly compounds in three steps of the investigated reaction: oxidation of aldehyde to peracid (redox function), activation of carbonyl group (Lewis acidity), and decomposition of the Criegee adduct to ε-caprolactone (Brønsted acidity).

## Figures and Tables

**Table 1:** Content of key elements in the investigated heteroply compounds determined by X-ray fluorescence (XRF).

Sample	W/Mo (wt. %)		P (wt. %)		Co/Mn/Fe (wt. %)	
	Calculated	Found	Calculated	Found	Calculated	Found
H_3_PW	98.6	98.6	1.4	1.4	-	-
H_3_PMo	97.4	97.4	2.6	2.6	-	-
H_2_Co_0.5_PW	97.3	97.3	1.4	1.5	1.3	1.2
HCoPW	96.1	96.2	1.3	1.4	2.6	2.4
Co_1.5_PW	94.9	95.11	1.3	1.4	3.8	3.4
H_2_Co_0.5_PMo	95.0	95.5	2.6	2.5	2.4	2.0
HCoPMo	92.8	93.2	2.5	2.3	4.8	4.5
Co_1.5_PMo	90.6	90.8	2.4	2.4	7.0	6.8
H_2_Mn_0.5_PW	97.4	97.5	1.4	1.4	1.2	1.1
HMnPW	96.3	96.5	1.3	1.4	2.4	2.1
Mn_1.5_PW	95.1	95.4	1.3	1.4	3.6	3.3
H_2_Mn_0.5_PMo	95.2	95.3	2.6	2.6	2.3	2.1
HMnPMo	93.0	93.3	2.5	2.6	4.5	4.2
Mn_1.5_PMo	91.0	91.3	2.5	2.4	6.5	6.3
H_2_Fe_0.5_PW	97.4	97.5	1.4	1.5	1.2	1.0
HFePW	96.2	96.4	1.4	1.4	2.4	2.3
Fe_1.5_PW	95.1	95.4	1.3	1.3	3.6	3.3
H_2_Fe_0.5_PMo	95.1	95.3	2.6	2.6	2.3	2.1
HFePMo	93.0	93.3	2.5	2.6	4.5	4.2
Fe_1.5_PMo	90.9	91.1	2.4	2.5	6.6	6.4

**Table 2:** Catalytic activity of the heteropoly salts in the dehydration of ethanol at 250 °C.

**Yield of (%)**	**H_3_PW**	**H_2_M_0.5_PW**	**HMPW**	**M_1.5_PW**	**H_3_PMo**	**H_2_M_0.5_PMo**	**HMPMo**	**M_1.5_PMo**
	**M = Co**				**M = Co**			
ethylene	95.9	97.8	49.0	20.3	30.0	14.1	12.5	8.2
diethyl ether	4.1	1.4	20.8	-	-	-	-	-
acetaldehyde	-	0.2	0.2	-	18.0	12.5	11.0	9.0
Conversion (%)	97.2	99.4	70.0	20.3	48.0	26.6	23.5	17.2
**Yield of (%)**	**H_3_PW**	**H_2_M_0.5_PW**	**HMPW**	**M_1.5_PW**	**H_3_PMo**	**H_2_M_0.5_PMo**	**HM_1_PMo**	**M_3_PMo**
	**M = Fe**				**M = Fe**			
ethylene	95.9	99.8	87.6	70.0	30.0	38.0	29.9	37.8
diethyl ether	4.1	-	-	-	-	-	-	-
acetaldehyde	-	-	-	-	18.0	9.1	10.7	22.7
Conversion (%)	97.2	99.8	87.6	70.0	48.0	47.1	40.6	60.5
**Yield of (%)**	**H_3_PW**	**H_2_M_0.5_PW**	**HMPW**	**M_1.5_PW**	**H_3_PMo**	**H_2_M_0.5_PMo**	**HM_1_PMo**	**M_1.5_PMo**
	**M = Mn**				**M = Mn**			
ethylene	95.9	99.9	99.5	20.4	30.0	42.0	7.2	10.6
diethyl ether	4.1	-	-	-	-	-	-	-
acetaldehyde	-	-	-	-	18.0	9.1	13.1	23.3
Conversion (%)	97.2	99.5	99.5	20.9	48.0	51.1	20.3	33.9

## Supplementary Materials

The following are available online at http://www.mdpi.com/1996-1944/11/7/1208/s1, Figure S1: FTIR spectra recorded in KBr of (a) H_3_PW, H_2_Co_0.5_PW, HCoPW, Co_1.5_PW, and (b) H_3_PMo, H_2_Co_0.5_PMo, HCoPMo, Co_1.5_PMo, Figure S2: FTIR spectra recorded in KBr of (a) H_3_PW, H_2_Mn_0.5_PW, HMnPW, Mn_1.5_PW, and (b) H_3_PMo, H_2_Mn_0.5_PMo, HMnPMo, Mn_1.5_PMo, Figure S3: FTIR spectra recorded in KBr of (a) H_3_PW, H_2_Fe_0.5_PW, HFePW, Fe_1.5_PW, and (b) H_3_PMo, H_2_Fe_0.5_PMo, HFePMo, Fe_1.5_PMo.
