# Supplementary material for: Keggin-Type Heteropoly Salts as Bifunctional Catalysts in Aerobic Baeyer-Villiger Oxidation
Source: Materials (Basel). 2018 Jul 13;11(7):1208. doi: 10.3390/ma11071208 (PMC6073195; doi:10.3390/ma11071208)
Supplement: Supplementary file 1 [file materials-11-01208-s001.pdf]

materials

MDPI

*Supplementary Materials*

# Keggin-Type Heteropoly Salts as Bifunctional Catalysts in Aerobic Baeyer-Villiger Oxidation

**Katarzyna Pamin [1,*], Jan Połtowicz [1], Mateusz Pronczuk [2], Joanna Kryściak-Czerwenka [1], Robert Karcz [1] and Ewa M. Serwicka [1]**

[1] Jerzy Haber Institute of Catalysis and Surface Chemistry, Polish Academy of Sciences, Niezapominajek 8, 30-239 Krakow, Poland; ncpoltow@cyf-kr.edu.pl (J.P.); nckrisci@cyf-kr.edu.pl (J.K.-C.); nckarcz@cyf-kr.edu.pl (R.K.); ncserwic@cyf-kr.edu.pl (E.M.S.)

[2] Faculty of Chemical Engineering and Technology, Cracow University of Technology, Warszawska 24, 31-155 Krakow, Poland; mpronczuk@chemia.pk.edu.pl

* Correspondence: ncpamin@cyf-kr.edu.pl; Tel.: +48-12-63-95-153

Received: 31 May 2018; Accepted: 9 July 2018; Published: date

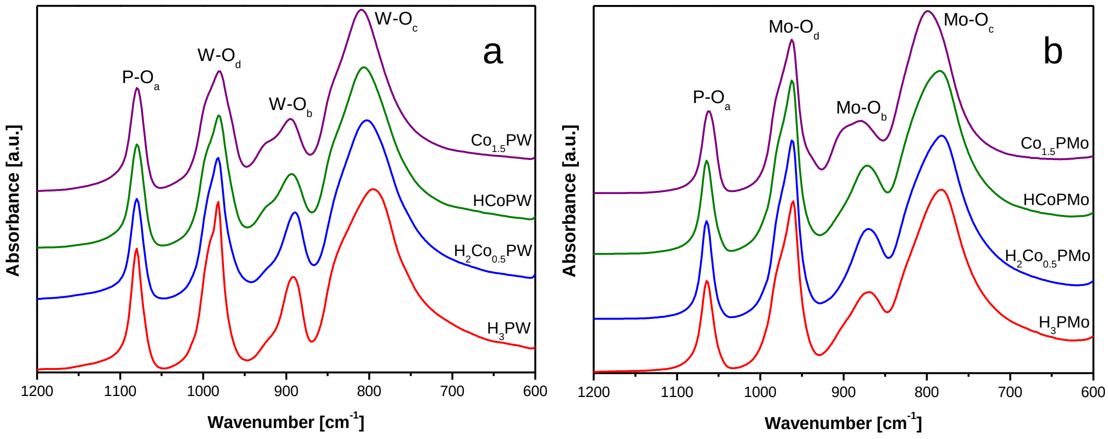


**Figure S1.** FTIR spectra recorded in KBr of (a) $H_3PW$, $H_2Co_{0.5}PW$, HCoPW, $Co_{1.5}PW$, and (b) $H_3PMo$, $H_2Co_{0.5}PMo$, HCoPMo, $Co_{1.5}PMo$.

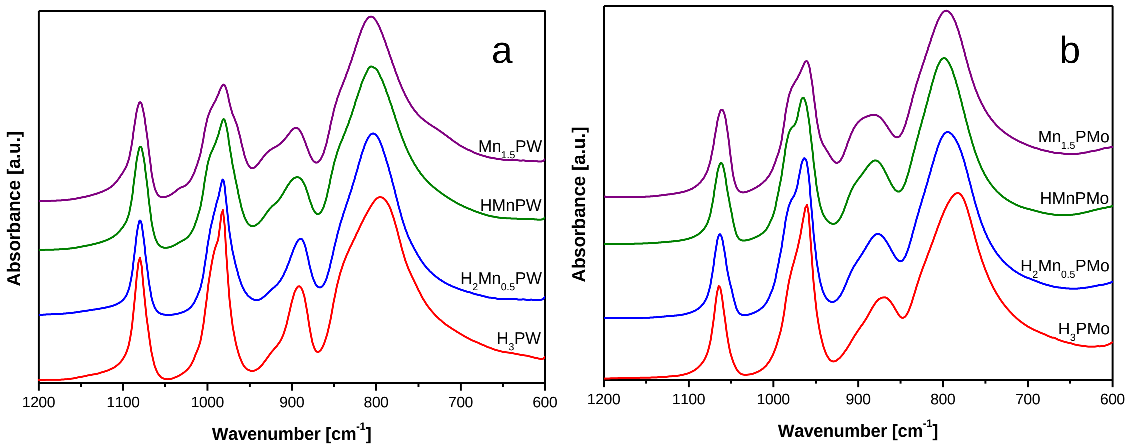


**Figure S2.** FTIR spectra recorded in KBr of (a) $H_3PW$, $H_2Mn_{0.5}PW$, HMnPW, $Mn_{1.5}PW$, and (b) $H_3PMo$, $H_2Mn_{0.5}PMo$, HMnPMo, $Mn_{1.5}PMo$.

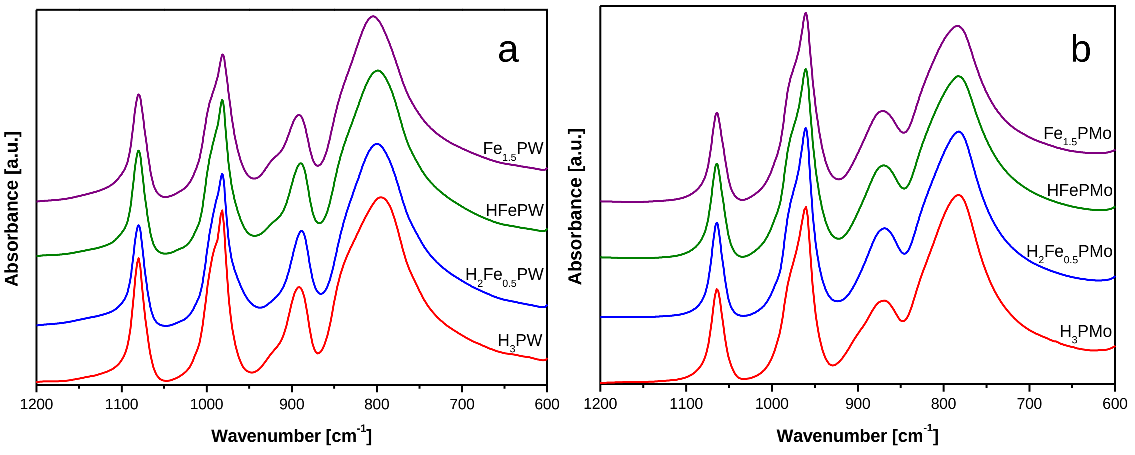


**Figure S3.** FTIR spectra recorded in KBr of (a) $H_3PW$, $H_2Fe_{0.5}PW$, HFePW, $Fe_{1.5}PW$, and (b) $H_3PMo$, $H_2Fe_{0.5}PMo$, HFePMo, $Fe_{1.5}PMo$.

© 2018 by the authors. Submitted for possible open access publication under the terms and conditions of the Creative Commons Attribution (CC BY) license (http://creativecommons.org/licenses/by/4.0/).
